# Correction: The interplay between dendritic cells and CD8 T lymphocytes is a crucial component of SARS-CoV-2 immunity

**DOI:** 10.1038/s41423-022-00916-8

**Published:** 2022-08-30

**Authors:** Jonas Buttenschön, Jochen Mattner

**Affiliations:** 1 Mikrobiologisches Institut - Klinische Mikrobiologie, Immunologie und Hygiene, Universitätsklinikum Erlangen and Friedrich-Alexander Universität (FAU) Erlangen-Nürnberg, Erlangen, Germany; 2 Medical Immunology Campus Erlangen, FAU Erlangen-Nürnberg, Erlangen, Germany

**Keywords:** Biomarkers, Immunology

Correction to: *Cellular & Molecular Immunology* 10.1038/s41423-020-00624-1, published online 08 January 2021

The licence information was missing from this article and should have been CC-BY.

The original article has been corrected.

